# Expressions of Kisspeptin System and *Ki‐67* in the Reproductive Tissues of Cyclic Bitches

**DOI:** 10.1111/rda.70205

**Published:** 2026-04-15

**Authors:** Özgecan Korkmaz Ağaoğlu, Ali Reha Ağaoğlu, Özlem Özmen, Gökhan Bozkurt, Şerife Taşan

**Affiliations:** ^1^ Department of Genetics, Faculty of Veterinary Medicine Burdur Mehmet Akif Ersoy University Burdur Türkiye; ^2^ Department of Obstetrics and Gynecology, Faculty of Veterinary Medicine Burdur Mehmet Akif Ersoy University Burdur Türkiye; ^3^ Department of Pathology, Faculty of Veterinary Medicine Burdur Mehmet Akif Ersoy University Burdur Türkiye

**Keywords:** canine estrus cycle, cell proliferation marker, immunohistochemistry, *KISS1*, *KISS1R*

## Abstract

Kisspeptin is a peptide that plays a pivotal role in the central regulation of gonadotropins. It is regarded as a key regulator of reproductive processes, including follicular and luteal development and endometrial remodelling. The objective of this study was to investigate the expression of *KISS1*, its receptor *KISS1R* and the proliferation marker *Ki‐67* in the uterus, oviduct and ovary of bitches at different stages of the oestrous cycle using reverse transcription quantitative polymerase chain reaction (RT‐qPCR) and immunohistochemistry. Thirty‐five healthy female dogs were divided into five groups based on reproductive stage as follows: anestrus, proestrus, estrus, diestrus and prepubertal. Each group consisted of an equal number of animals (*n* = 7). *KISS1* mRNA expression was significantly elevated in estrus, while *KISS1R* mRNA expression remained consistently low across all tissues. *Ki‐67* mRNA expression increased significantly in the uterus during estrus, whereas the highest levels in the oviduct and ovary were observed in anestrus. Immunohistochemical analysis revealed a marked increase in *KISS1* expression in the endometrium during estrus, accompanied by elevated *Ki‐67* positivity in the uterus, oviduct and ovary, particularly in glandular epithelial and luteal cells. Conversely, *KISS1R* protein showed strong localisation during estrus despite low transcript levels, suggesting possible post‐transcriptional regulation. The synchronised upregulation of *KISS1* and *Ki‐67* in the endometrium during estrus supports their coordinated roles in endometrial proliferation and remodelling. These findings emphasise the significance of local kisspeptin signalling in canine reproductive tissues and suggest its contribution to tissue adaptations during the estrous cycle. The results provide new insights into the peripheral functions of the kisspeptin system and highlight its potential as a target for improving reproductive management in bitches.

## 1 Introduction

Sustainable dog breeding requires not only optimal care and management practices but also a thorough understanding and control of reproductive efficiency. One of the key components in achieving sustainable breeding outcomes is maintaining consistently high litter productivity. Achieving this goal necessitates detailed knowledge of reproductive physiology, including the oestrous cycle, pregnancy and the postpartum period, as well as the underlying mechanisms that regulate these processes. Such understanding enables the development of advanced reproductive management strategies or therapeutic strategies for infertility. One of the fundamental systems involved in regulating these reproductive events is the hypothalamic–pituitary–gonadal (HPG) axis. This axis orchestrates hormone secretion to maintain reproductive homeostasis in mammals. The HPG axis functions primarily through the release of gonadotropin‐releasing hormone (GnRH) from the hypothalamus, which in turn regulates the secretion of follicle‐stimulating hormone (FSH) and luteinising hormone (LH) from the anterior pituitary (Zeydabadi Nejad et al. 2017). These gonadotropins act on the gonads to promote gametogenesis and the production of steroid hormones.

GnRH secretion is modulated by several neuropeptides and neurotransmitters, such as leptin (Quennell et al. 2011), dopamine (Liu and Herbison 2013) and kisspeptin (Iwata et al. 2019). Among these, kisspeptin has emerged as a central regulator of the HPG axis and it was shown to play a crucial role in reproductive hormone regulation by stimulating GnRH neurons (Ohtaki et al. 2001). The kisspeptin gene (*KISS1*) is expressed not only in the anteroventral periventricular nucleus of the brain but also in peripheral reproductive organs, including the placenta, ovaries and testes (Bhattacharya and Babwah 2015; Uenoyama et al. 2016).

The presence of *KISS1* and its receptor in reproductive tissues has been confirmed in various species, including rats (Terao et al. 2004), hamsters (Shahed and Young 2009), dogs (Schäfer‐Somi et al. 2017), cats (Tanyapanyachon et al. 2018; Cardoso Santos et al. 2022) and humans (Gaytán et al. 2009). These studies indicate that *KISS1* plays a role not only in central hormonal regulation but also in peripheral reproductive processes, influencing follicular dynamics, luteal development and endometrial remodelling. Cell proliferation is essential for follicular and luteal development as well as for endometrial remodelling. Recent studies demonstrated that exogenous KP‐10 administration significantly increased gonadotropin secretion and modulated ovarian function in bitches (Bozkurt et al. 2026). Previous studies have focused on physiological contexts, such as pregnancy or exogenous peptide administration and have not concentrated on oestrous cycle dependent expression patterns of *KISS1* and *KISS1R* across multiple reproductive tissues. Moreover, the potential association between local *KISS1* signalling and tissue‐specific proliferative dynamics has not been systematically researched in cyclic bitches. Given that coordinated tissue remodelling during the oestrous cycle depends on tightly regulated cellular proliferation, evaluating a reliable marker of mitotic activity may provide additional functional insight. *Ki‐67*, a nuclear protein expressed in all active phases of the cell cycle except G0 (Trihia et al. 2003), has been shown to exhibit sexual cycle stage dependent variation in reproductive tissues of mares (Aupperle et al. 2000), cows (Arai et al. 2013) and bitches (Van Cruchten et al. 2004).

In light of these findings, the current study aims to quantify the expression of *KISS1*, *KISS1R* and *Ki‐67* in the ovaries, oviducts and uterus of bitches at different stages of the oestrous cycle. By characterising their spatial and temporal expression profiles, this study seeks to clarify the extent to which *KISS1* signalling is involved in regulating tissue‐specific proliferation and overall reproductive competence.

## 2 Materials and Methods

### 2.1 Experimental Design

In this study, 35 healthy female dogs, aged 2–3 years, were used, which were brought to the Burdur MAKUVET Animal Hospital by their owners for spaying. The bitches underwent routine clinical, gynaecological examinations and preanesthetic blood analysis were performed. Based on the medical history information obtained from the owners of the dogs determined to be healthy after the examinations, vaginal smear samples were taken from bitches that had previously shown estrus at least once (*excluding group 5/prepubertal bitches) to determine which stage of the estrus cycle they were in (Reckers et al. 2022). Oestrous cycle stage determination was performed using vaginal cytology assessment, in accordance with the standard pre‐spaying clinical protocol of the Animal Hospital. As the systemic hormonal analyses were not in the standard protocol, hormone measurements were not performed. After the examination, the bitches were divided into 5 groups: anestrus (G1), proestrus (G2), estrus (G3), diestrus (G4) and prepubertal stage (G5). This study was approved by the Burdur MAKU Local Ethics Committee on Animal Experiments (Approval no: 650 and 1308).

### 2.2 Vaginal Cytology Evaluation

The percentages of cell types in vaginal cytological slides were calculated to determine the stage of the estrus cycle (Table 1).

**TABLE 1:** Determination of estrous cycle stages based on vaginal cytology in bitches (Kubicek 1978).

Estrous cycle stage	Cell type	Cell distribution (%)
Anestrus	Parabasal and small intermediate cells	Parabasal: ~70%–90% Intermediate: ~10%–30% Superficial: 0%
Proestrus	Large intermediate and superficial cells	Superficial: ~40%–60% Intermediate: ~30%–50% Parabasal: ~10%
Estrus	Predominantly superficial cells (many anuclear)	Superficial: ≥ 80% Aneukaryotic: Common Others: < 10%
Diestrus	Intermediate and parabasal cells	Intermediate: ~50%–70% Parabasal: ~20%–40% Superficial: < 10%
Prepubertal stage	Parabasal cells	Parabasal: > 90%

### 2.3 Tissue Sampling

After the ovariohysterectomy operation, tissues were dissected on a sterile cutting board using a sterile scalpel and forceps. Two sections were collected from the uterus, oviduct and ovaries (including corpus luteum/CL; corpus albicans/CA and follicles). Ovarian samples were sectioned horizontally into two parts; uterine samples were obtained transversely from the median part of the right uterine horn, including all tissue layers and the right oviduct was bisected transversely. One portion of each sample was wrapped in aluminium foil, rapidly frozen in liquid nitrogen, transported under appropriate conditions to the laboratory and stored at −86°C until RNA isolation processing. After that, the second portion of each sample was placed in labelled tissue cassettes and immediately fixed in 10% neutral buffered formaldehyde. Following fixation, they were transported to the laboratory, where routine histological processing was performed and samples were embedded in paraffin blocks. H&E‐stained sections were assessed under light microscopy by two blinded and experienced histopathologists.

### 2.4 Immunohistochemistry (IHC)

The primary antibodies used were KISS1 (Anti‐KISS‐1 antibody 24‐Q, sc‐101,246) (Santa Cruz, Texas, USA), KISS1R (Anti‐GPR54 antibody—C‐terminal, ab188995) (Abcam, Cambridge, UK) and Ki‐67 (Polyclonal antibody, 27309‐1‐AP) (Proteintech, IL, USA), all diluted 1:100 in antibody dilution buffer. Immunohistochemistry was performed using a streptavidin–biotin–peroxidase method. Slides were deparaffinised in xylene, rehydrated through graded ethanol and rinsed in distilled water. Antigen retrieval was performed by heating the slides twice for 5 min in citrate buffer (pH 6.0) using a microwave oven. Endogenous peroxidase activity was blocked with 3% hydrogen peroxide in methanol for 20 min. Following. After two 10‐min PBS washes, the slides were incubated with normal (goat) serum for 15 min to block nonspecific binding. Primary antibodies were applied overnight at +4°C. The next day, slides were washed in PBS and incubated with biotin‐labelled serum for 30 min, followed by streptavidin‐horseradish peroxidase for 30 min, with PBS washes between steps. Immunoreactivity was visualised using freshly prepared DAB (3,3‐diaminobenzidine) chromogen, and detection was completed using the Mouse and Rabbit Specific HRP/DAB Detection Kit—Micropolymer (ab236466) (Abcam, Cambridge, UK). Slides were counterstained with Harris haematoxylin, dehydrated, cleared and mounted in Entellan. Negative controls were processed identically except that the primary antibody was replaced with antibody diluent, and all other steps remained unchanged. All evaluations were independently conducted by two blinded histopathologists.

The quantification of immunostaining was performed by counting 20 cells in each of five randomly selected microscopic fields (a total of 100 cells per sample) at 40× magnification. The number of immunopositive cells was analysed using ImageJ software (version 1.48; NIH, Bethesda, MD, USA). Photographs were taken with an Olympus CX41 microscope, and morphometric analyses were conducted using CellSens Life Science Imaging Software (Olympus Corporation, Tokyo, Japan).

### 2.5 RT‐qPCR

Total RNA was extracted from ovarian (including corpus luteum and corpus albicans when present), uterine and oviductal tissues using Trizol reagent according to the manufacturer's instructions. Following RNA isolation, RNA quality and quantity were assessed. RNA integrity was evaluated via 0.8% agarose gel electrophoresis. Concentration and purity were measured using a NanoDrop ND‐2000 spectrophotometer (Thermo Fischer Scientific, Wilmington, DE). Based on the quantification results, RNA samples were diluted and subjected to ethanol precipitation. DNA digestion and cDNA synthesis were performed according to the manufacturer's instructions for the commercial kit (Thermo Fischer Scientific, Wilmington, DE). DNAse I (Thermo Fisher Scientific, Wilmington, DE) was used for digestion to eliminate DNA contamination.

Primer sets for the target genes (*KISS1*, *KISS1R* and *Ki‐67*) and 3 different housekeeping genes (HKGs) were identified through database searches (GenBank, NCBI) and/or designed using Primer3, IDT PrimerQuest and NCBI Primer‐BLAST tools (Table 2). Primer selection for RT‐qPCR analysis was carefully optimised to avoid non‐specific amplification. To verify primer specificity, melting curve analysis and agarose gel electrophoresis were performed in parallel.

**TABLE 2:** List of genes and nucleotide sequences.

Gene names	Sequence (5′→3′)	Product length (bp)	Accession number
Dog‐TUBA1	F: 5′‐GCCCTACAACTCCATCCTGA‐3′ R: 5′‐ATGGCCTCGTTGTCCACCA‐3′	78	XM_022420785.1
Dog‐HPRT1	F: 5′‐GAGATGTGATGAAGGAGATG‐3′ R: 5′‐CGACCAAGGAAAGCAAGGT‐3′	300	XM_035720782.1
Dog‐HMBS	F: 5′‐GACTCTGCTTCGCTGCATT‐3′ R: 5′‐AGGTACAGTTGCCCATCCTT‐3′	108	XM_038664685.1
Dog‐KISS1	F: 5′‐TGGTTTCTTGGCAGCTAATGC‐3′ R: 5′‐TGCATACCTGCAGGTCCAGG‐3′	100	XM_038448758.1
Dog‐KISS1R	F: 5′‐GCCACAAGCAGATGCGGA‐3′ R: 5′‐GGCACGCAGCACAGCAG‐3′	85	XM_035703419.1
Dog‐Ki‐67	F: 5′‐CCCACCTGTCCTGAAGAAAA‐3′ R: 5′‐TGTGGTCACTTCCAGTTGGTT‐3′	88	XM_038440934.1

### 2.6 Statistical Analysis

RT‐qPCR data were quantified using Roche Nano LightCycler software. Three genes, *HMBS*, *HPRT1* and *TUBA1*, were selected as references for the normalisation of target gene expression. Normalisation was performed using the 2^−ΔΔCt^ method described by Livak and Schmittgen (2001). Quantitative expression levels of *KISS1*, *KISS1R* and *Ki‐67* were calculated relative to the selected reference genes.

All statistical analyses of the *RT‐qPCR* and immunohistochemical data were conducted using Minitab software. Firstly, the data were tested for normality. As the data did not exhibit a normal distribution, the non‐parametric Kruskal–Wallis test was employed instead. For each gene and group, mean, median and standard deviation values were calculated. Box plot graphs were then generated to visualise the gene expression patterns.

The one‐way ANOVA test was performed to compare the immunohistochemical data among the groups. Post hoc differences were evaluated using Duncan's test, with *p*‐values < 0.05 considered statistically significant.

## 3 Results

### 3.1 Expression Profiles of KISS1/KISS1R and Ki‐67

**FIGURE 1:**
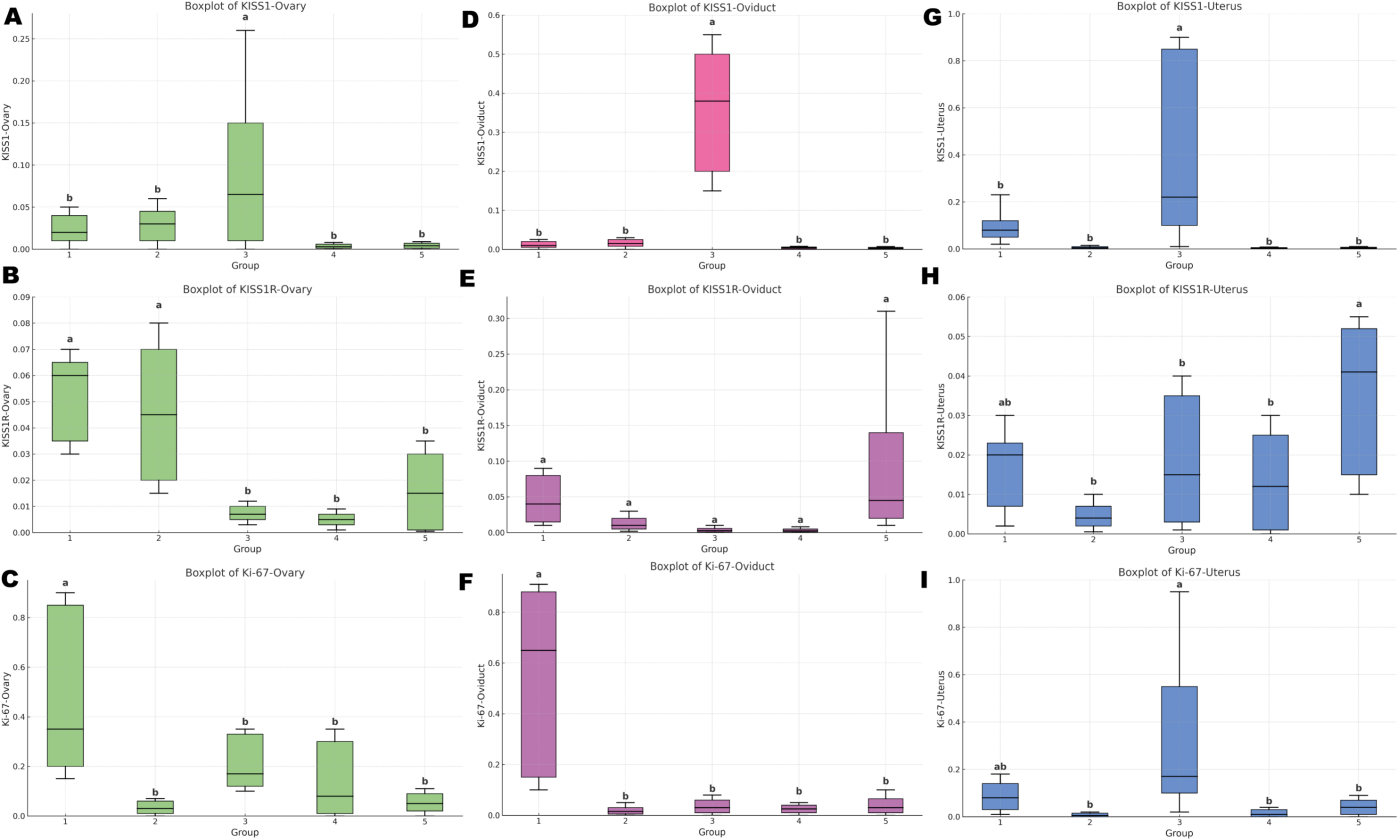
Relative mRNA expression levels of *KISS1* (A–C), *KISS1R* (D–F) and *Ki‐67* (G–I) in ovarian (A, D, G), oviductal (B, E, H) and uterine (C, F, I) tissues across different stages of the estrous cycle. Green panels represent ovarian tissue, pink panels represent oviductal tissue and blue panels represent uterine tissue. Groups: G1, anestrus; G2, proestrus; G3, estrus; G4, diestrus; G5, prepubertal. Expression levels were normalised to reference genes and calculated using the ΔCt method. Boxes indicate median and interquartile range; whiskers represent minimum and maximum values. Different letters above the boxplots indicate statistically significant differences between groups (*p* < 0.05).

Expression levels show differences among tissues and estrus cycle stages (Figure 1). *KISS1* expression level showed an increase during estrus (G3) in uterine (Figure 1C) and oviductal tissues (Figure 1B), whereas ovarian expression (Figure 1A) exhibited a moderate elevation in G3 compared with other groups. In contrast, *KISS1* expression remained relatively low in the remaining stages across all tissues.

*KISS1R* mRNA levels displayed a different pattern. In ovarian tissue (Figure 1D), higher expression was observed in G1 and G2, followed by a decline in G3 and G4. In uterine tissue (Figure 1F), expression was comparatively higher in G5, whereas oviductal expression (Figure 1E) remained low in most groups, with a noticeable increase in G5. Regarding *Ki‐67*, uterine expression (Figure 1I) peaked during estrus (G3), while ovarian (Figure 1G) and oviductal (Figure 1H) tissues showed their highest values in G1. These findings indicate that transcriptional regulation of the kisspeptin system and proliferative activity varies according to both tissue type and estrus cycle stage.

### 3.2 Histopathology and Immunohistochemistry

**FIGURE 2:**
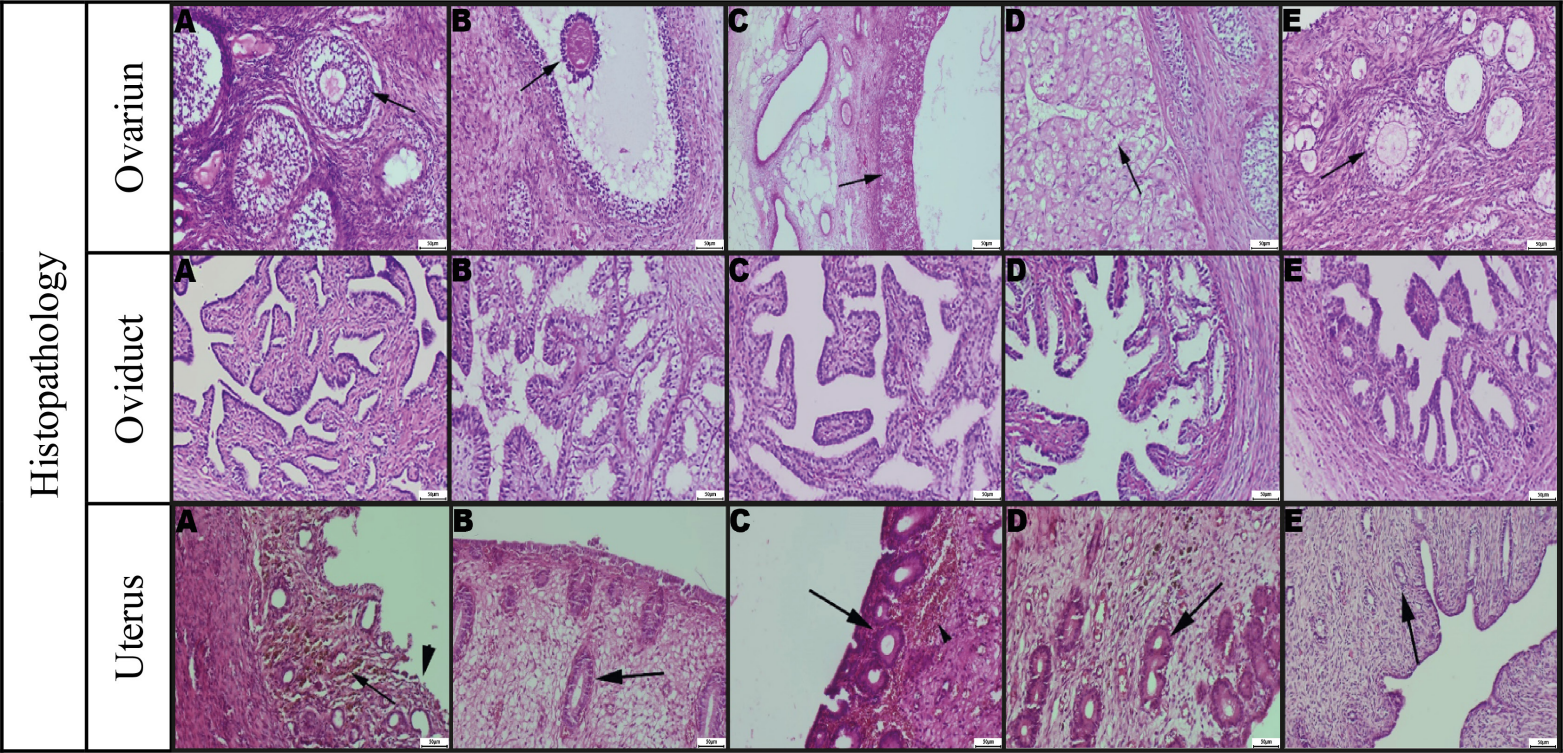
Ovarium: (A) cystic follicles and primary follicles (arrow), (B) A tertiary follicle (arrow), (C) A follicle undergoing ovulation (arrow), (D) Corpus luteum (arrow), (E) primordial follicles (arrow). Oviduct: (A) Atrophic appearance of the epithelium and increased connective tissue, (B) Epithelial proliferation and mild proteinaceous exudate in the lumen, (C) Hyperemia in blood vessels and regular epithelial morphology, (D) Epithelial degeneration and shedding, (E) Appearance of thin and short crypts. Uterus: (A) Epithelial shedding (thick arrowhead) and presence of hemosiderin pigment (thin arrow), (B) Glandular proliferation (thick arrow), (C) Glandular proliferation (thick arrow) and haemorrhage (thin arrowhead), (D) Glandular proliferation (thick arrow), (E) Atrophic glands (thick arrow). [A: Anestrus; B: Proestrus; C: Estrus; D: Diestrus; E: Prepubertal stage]. H&E, Bar: 50 μm.

No pathological findings were observed in the histopathological evaluation performed prior to the immunohistochemical examination (Figure 2).

**FIGURE 3:**
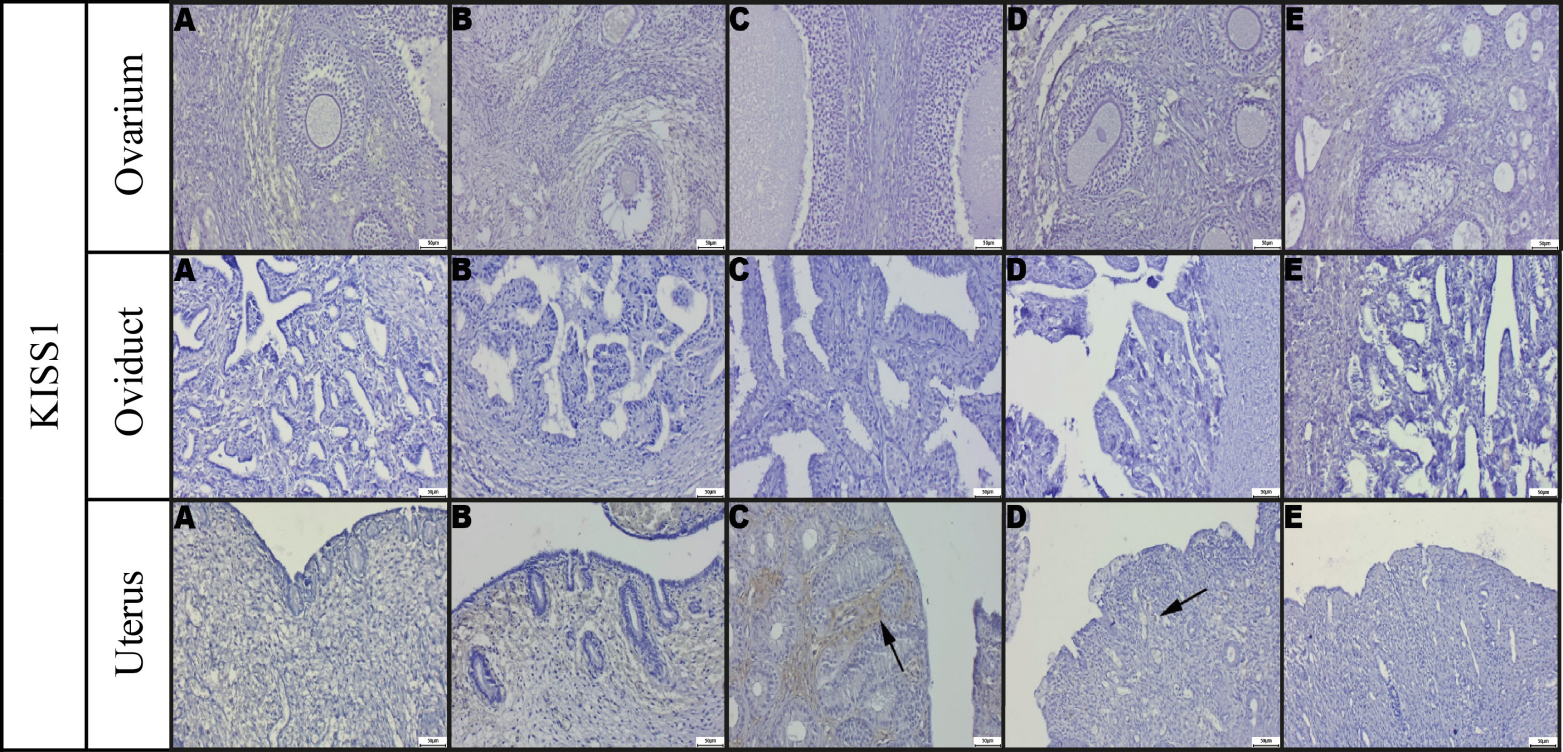
Immunohistochemical staining of KISS1. Ovarium: (A), (B), (C), (D) and (E) negative and/or weak staining. Oviduct: (A), (B), (C), (D) and (E) negative and/or weak staining. Uterus: (A) Negative and/or weak staining, (B) Negative and/or weak staining, (C) Markedly increased staining (arrow), (D) Mild expression in a few cells (arrow), (E) Weak expression (arrow). [A: Anestrus; B: Proestrus; C: Estrus; D: Diestrus; E: Prepubertal stage]. Streptavidin–biotin–peroxidase, Bar: 50 μm.

**FIGURE 4:**
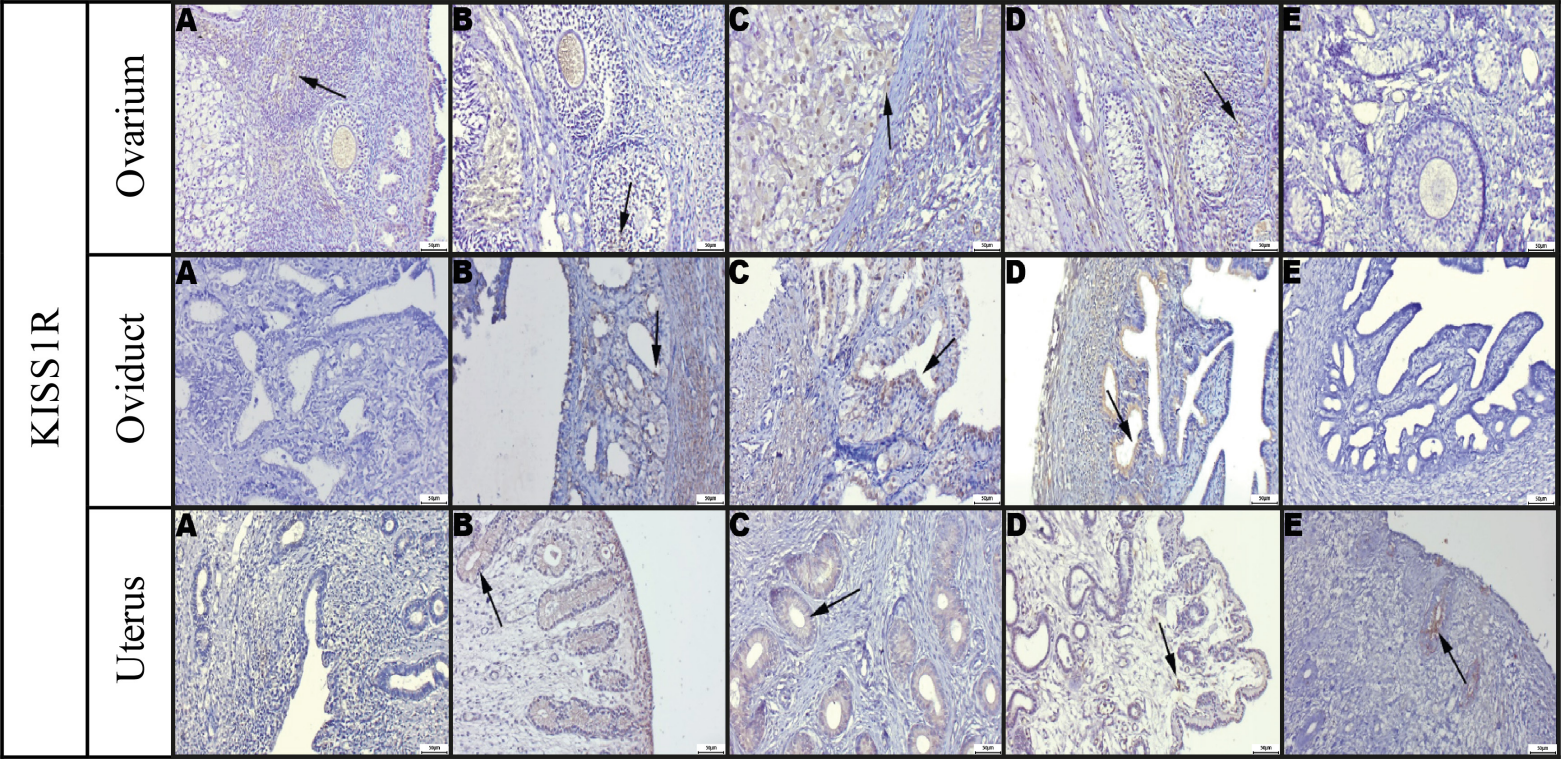
Immunohistochemical staining of KISS1R. Ovarium: (A) Mild staining (arrow), (B) Slightly increased staining (arrow), (C) Increased immunoreactivity (arrow), (D) Mild staining (arrow), (E) Negative and/or weak staining. Oviduct: (A) Negative or weak staining, (B) Slight staining (arrow), (C) Markedly increased staining (arrow), (D) Mild staining (arrow), (E) Negative or weak staining. Uterus: (A) Negative or weak staining, (B) Slightly increased staining in glandular epithelium (arrow), (C) Markedly increased staining in glandular epithelium (arrow), (D) Mild staining (arrow), (E) Mild staining (arrow). [A: Anestrus; B: Proestrus; C: Estrus; D: Diestrus; E: Prepubertal stage]. Streptavidin‐biotin‐peroxidase, Bar: 50 μm.

**FIGURE 5:**
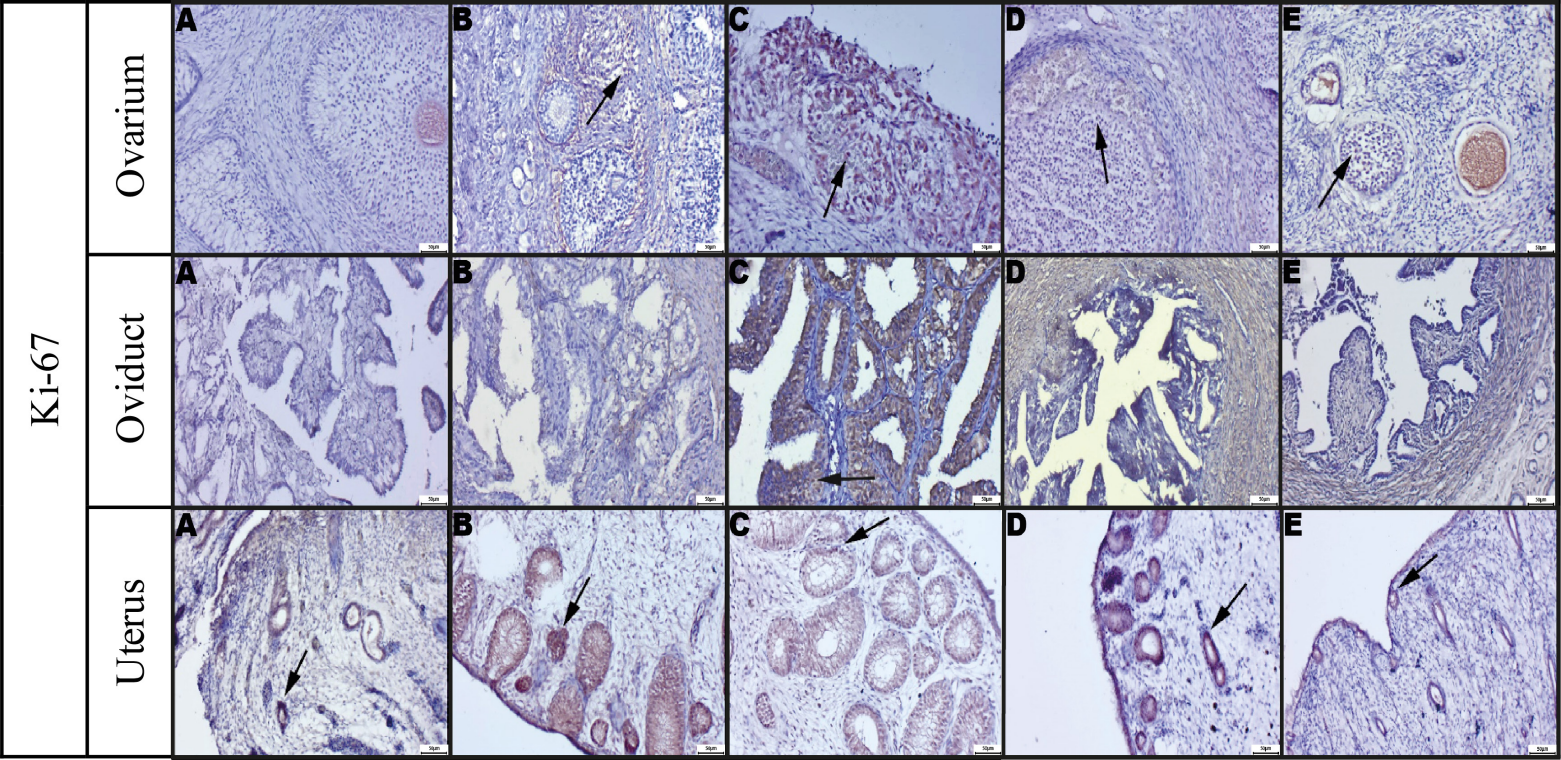
Immunohistochemical staining of Ki‐67. Ovarium: (A) Very weak staining, (B) Increased staining (arrow), (C) Markedly increased staining (arrow), (D) Substantially decreased staining (arrow), (E) Negative or weak staining. Oviduct: (A) Negative and/or weak staining, (B) Negative and/or weak staining, (C) Markedly increased staining (arrow), (D) Very weak staining in a few cells, (E) Negative or weak staining. Uterus: (A) Very weak staining (arrow), (B) Markedly increased staining, (C) Prominent staining (arrow), (D) Decreased staining (arrow), (E) Very weak staining (arrow). [A: Anestrus; B: Proestrus; C: Estrus; D: Diestrus; E: Prepubertal stage]. Streptavidin–biotin–peroxidase, Bar: 50 μm.

Immunohistochemical analysis revealed that KISS1 expression remained relatively stable in the ovary and oviduct across different stages, but it showed a noticeable increase in the uterus during estrus (Figure 3). In contrast, KISS1R expression was most prominent during estrus in the uterus, oviduct and ovary (Figure 4). Ki‐67 expression, which reflects proliferative activity, was also found to be significantly elevated in the uterus, oviduct and ovary during estrus (Figure 5). Qualitative evaluation demonstrated marker‐specific localisation patterns. KISS1 and KISS1R immunoreactivity was predominantly cytoplasmic in epithelial and luteal cells, whereas *Ki‐67* staining was strictly nuclear and confined to proliferating cells. Stromal areas generally exhibited weak or absent staining for KISS1 and KISS1R. Staining intensity varied according to oestrus cycle stage, with the strongest and most diffuse labelling observed during proestrus and estrus. In the ovaries, immunopositivity was mainly detected in preantral and antral follicles and particularly in the corpora lutea, where large luteal cells showed the strongest staining. In contrast, the ovarian stromal regions exhibited minimal or no staining. When comparing corpora lutea in diestrus with corpora albicans in anestrus, higher expression levels of all markers were consistently observed in the corpora lutea (Figures 3, 4, 5).

The findings obtained through quantitative evaluation of immunopositive cells, based on counts from representative microscopic fields, are summarised in the following table (Table 3).

**TABLE 3:** Quantification of immunopositive cells.

Group	KISS1 (Uterus)	KISS1R (Uterus)	Ki‐67 (Uterus)	KISS1 (Oviduct)	KISS1R (Oviduct)	Ki‐67 (Oviduct)	KISS1 (Ovary)	KISS1R (Ovary)	Ki‐67 (Ovary)
G1	1.00 ± 1.00ᵃ	1.42 ± 1.39ᵃ	11.85 ± 2.34ᵃ	0.42 ± 0.20ᵃ	1.42 ± 1.39ᵃ	8.00 ± 2.00ᵇ	1.42 ± 1.13ᵃᵇ	14.71 ± 2.56ᵇ	6.57 ± 1.27ᵃ
G2	3.14 ± 1.06ᵇ	16.00 ± 2.70ᶜ	61.00 ± 2.30ᶜ	1.71 ± 0.95ᵇ	20.71 ± 2.49ᶜ	15.57 ± 1.71ᶜ	1.28 ± 1.11ᵃ	29.42 ± 2.93ᶜ	21.00 ± 2.23ᶜ
G3	15.50 ± 3.14ᶜ	31.66 ± 2.80ᵈ	60.83 ± 2.71ᶜ	4.50 ± 1.22ᶜ	46.16 ± 5.56ᵈ	43.50 ± 3.01ᵈ	2.66 ± 1.36ᵇ	46.00 ± 4.00ᵈ	32.50 ± 3.39ᵈ
G4	0.85 ± 0.34ᵃ	12.57 ± 2.14ᵇ	35.14 ± 1.95ᵇ	0.57 ± 0.29ᵃ	12.57 ± 2.14ᵇ	14.28 ± 1.70ᶜ	1.14 ± 0.89ᵃ	13.00 ± 5.35ᵇ	21.42 ± 2.29ᶜ
G5	0.28 ± 0.18ᵃ	2.00 ± 1.82ᵃ	10.85 ± 1.34ᵃ	0.28 ± 0.18ᵃ	0.71 ± 0.28ᵃ	1.14 ± 0.89ᵃ	1.00 ± 0.43ᵃ	1.85 ± 1.77ᵃ	10.42 ± 1.27ᵇ
*p*	< 0.001	< 0.001	< 0.001	< 0.001	< 0.001	< 0.001	< 0.001	< 0.001	< 0.001

*Note:* Different letters in the same column indicate differences between groups.

## 4 Discussion

In this study, the expression of *KISS1*, *KISS1R* and *Ki‐67* in the uterine, oviductal and ovarian tissues of bitches during the oestrous cycle was investigated. The results of the study show that the gene expressions in the examined tissues exhibit variation according to the stage of oestrous cycle, reflecting dynamic regulatory mechanisms. To better understand the functional relevance of these genes, previous studies in various species provide valuable insights. This finding demonstrates that, as reported in various studies conducted in species such as cats (Tanyapanyachon et al. 2018; Cardoso Santos et al. 2022), rats (Castellano et al. 2006), hamsters (Shahed and Young 2009) and dogs (Cielesh et al. 2017; Schäfer‐Somi et al. 2017), *KISS1/KISS1R* exerts cellular‐level effects on the uterus and ovary and *Ki‐67* plays an active role in the regulation of the changes occurring in the uterus and ovaries throughout the oestrous cycle.

In a study conducted in mice, it was determined that endometrial *KISS1/KISS1R* mRNA expression levels remained low during the first four days of pregnancy. With the formation of decidualisation, an increase in *KISS1/KISS1R* expression was observed, leading to the conclusion that *KISS1* and its receptor play a functional role during decidualisation (Zhang et al. 2014). Building on these findings, the current study aims to explore similar expression patterns in bitches throughout the oestrous cycle. In analyses of uterine tissue obtained from cats at different stages of the oestrous cycle, *KISS1* was found to be more highly expressed in diestrus than in other stages, while *KISS1R* showed increased expression in proestrus and estrus. Moreover, a positive correlation between uterine *KISS1* and *KISS1R* expression levels during the oestrous cycle was identified (Cardoso Santos et al. 2021). In goats, studies showed no differences in oviductal *KISS1* expression between breeding and anestrus seasons. Meanwhile, in breeding season goats, there was no significant difference in mRNA expression between uterus and oviduct tissues, but in anestrus goats, *KISS1* expression in the oviduct was higher compared to the uterus (Samir et al. 2024). In this study, uterine *KISS1* expression peaked during estrus, when endometrial proliferation was most pronounced. These molecular observations raise important questions about their physiological implications, particularly in relation to endometrial remodelling. This suggests that *KISS1* may be involved in estrus‐related endometrial thickening, glandular growth and secretory activation. *Ki‐67* expression appears to be associated with physiological cell turnover accompanying normal tissue growth.

Studies on the effects of the *KISS1* gene on the reproductive system have shown that in rats, *KISS1* and *KISS1R* genes are expressed in the ovary. While *KISS1R* expression did not vary significantly across oestrous cycle stages, *KISS1* was expressed at low levels at the beginning of proestrus, showed a marked rise in late proestrus, then a sudden decrease in estrus and thereafter maintained low but constant expression across other stages (Castellano et al. 2006). Such patterns align with what has been reported in other mammals, reinforcing the evolutionary conservation of these regulatory pathways. In another study in cats, ovarian *KISS1* expression was higher during the follicular phase than the luteal phase (Tanyapanyachon et al. 2018). Another study found that *KISS1* and *KISS1R* expression in the ovaries of diestrus was higher than in anestrus in cats (Cardoso Santos et al. 2022). This study also found that *KISS1* expression was significantly higher during the estrus stage, when follicles are at their most developed, than at other times, suggesting that *KISS1* plays a regulatory role in canine follicular growth.

From an immunohistochemical perspective, the findings of the study can be interpreted as follows. During the oestrous cycle in bitches, various hormones are secreted, causing multiple changes in the genital organs and the mucosa lining their lumens. For example, rapid follicular development during proestrus is accompanied by increased oestrogen secretion from the follicles. These oestrogens stimulate mitotic division in luminal endometrial cells from proestrus through early estrus. From the second half of estrus, endometrial mitotic rate slows due to decreased numbers of oestrogen and progesterone receptors in endometrial cells. In late estrus and early diestrus, following preovulatory luteinisation and ovulation, increasing progesterone secretion coincides with glandular proliferation. During diestrus, elevated progesterone levels suppress oestrogen‐mediated proliferative activity and promote differentiation and secretory transformation of the endometrium. Consequently, the endometrial mitotic rate remains low during this stage. In anestrus, decreased serum progesterone and oestrogen levels are associated with reductions in endometrial thickness and gland number (Van Cruchten 2004). These cyclical changes in the canine endometrium prepare the uterus for potential pregnancy after estrus. If pregnancy does not occur, the endometrium reverts to its previous state and enters anestrus.

The *KISS1* gene has also been shown to act as a regulatory factor in all these endometrial events. For example, in mice, *KISS1* and *KISS1R* knockout animals exhibited 93% fewer uterine glands compared to normal mice and uterine tissue sections were on average 70% smaller (León et al. 2016). In women, KISS1/KISS1R expression was observed mainly in endometrial epithelial and stromal cells but not in myometrial cells, indicating a role in morphological changes in the uterus during the menstrual cycle (Roman et al. 2012). In goats, *KISS1* was expressed in the uterus and oviduct samples from both breeding‐season and anestrus animals. In these goats, KISS1 was strongly localised in glandular and luminal epithelium, weakly in vascular and stromal epithelial cells and moderately in myometrial cells. Moreover, staining intensity of KISS1 was greater in anestrus goats than those in breeding season (Samir et al. 2024). A study in cats showed that KISS1 and KISS1R were expressed in luminal and glandular epithelium of the uterus throughout the follicular and luteal phases, with KISS1 also expressed in stromal cells, but KISS1R was not expressed there. Immunostaining for both in the myometrium was positive, though vascular cells were negative for KISS1R (Tanyapanyachon et al. 2018). Endometrial and glandular epithelial cells play important roles in synthesising and secreting nutrients necessary for embryo or fetus survival and development (Filant and Spencer 2014). Thus, glandular development and functional differentiation are critical for embryo survival (Spencer 2014). This study showed that KISS1 and KISS1R are differentially localised in uterine tissues across the oestrous cycle, appearing in the luminal and stromal epithelium during proestrus and shifting to the glandular epithelium during estrus, which supports their regulatory roles in adenogenesis and endometrial remodelling. The concurrent expression of *Ki‐67* with *KISS1* and *KISS1R* further suggests that the kisspeptin system is correlated with mitotic activity, suggesting a modulatory or permissive role in endometrial proliferation and differentiation.

During the canine oestrous cycle, the endometrium undergoes numerous morphological, cellular and biochemical changes (Van Cruchten 2004). In parallel, the role of *Ki‐67* as a marker of cellular proliferation provides further support for these interpretations. Studies have demonstrated roles for steroid hormone receptors (Vermeirsch et al. 2000), matrix metalloproteinases (Chu et al. 2002) and apoptosis (Chu et al. 2006) in orchestrating these cyclical changes. However, detailed and up‐to‐date information on proliferative patterns in canine cyclic endometrium is scarce. For example, it was found that endometrial gland growth occurred from the end of anestrus to the end of estrus in bitches (Barrau et al. 1975). Another study noted proliferation in all endometrial cells during proestrus in cyclic bitches (Spanel‐Borowski et al. 1984). A recent study in sheep reported that *Ki‐67* expression was higher in the follicular and early luteal stages compared with other stages of the oestrous cycle (Benbia et al. 2020). In bitches, it was shown that the number of Ki‐67 positive cells in the endometrium was higher during proestrus than during the early and late diestrus. Moreover, these cells were mainly found in the stroma and luminal epithelium, with fewer cells found in the glands and more in the blood vessels. During estrus, the number of positive cells in vascular sites decreased while the luminal count remained high, with the greatest number found in glandular epithelial cells. During diestrus and anestrus, the number of positive cells dropped significantly in stromal, luminal and glandular epithelial cells (Van Cruchten et al. 2004). In rats, it was found that proestrus and estrus featured many Ki‐67 positive cells in the myometrium and stroma, fewer in glandular epithelial cells; while in diestrus localisation remained but with reduced intensity (Marusak et al. 2007). This study confirms that increased Ki‐67 expression in endometrial epithelial cells during proestrus and estrus indicates high proliferative activity in these stages.

In conclusion, the synchronisation of *KISS1* and *Ki‐67* expression during estrus, especially in the uterus, suggests a coordinated role in endometrial remodelling and cell proliferation. Interestingly, although *KISS1R* mRNA expression remained consistently low across all examined tissues and oestrous cycle stages, immunohistochemical analysis revealed strong protein localisation in the uterus, oviduct and ovary, particularly during estrus. This apparent discrepancy between transcript and protein levels suggests that *KISS1R* expression in canine reproductive tissues may be regulated through post‐transcriptional mechanisms. Potential explanations include mRNA stabilisation, differential translation efficiency, or reduced mRNA degradation, all of which can result in elevated protein expression despite low transcript abundance. Alternatively, increased protein stability or accumulation in specific tissue compartments during estrus may also contribute to this phenomenon. Such regulation aligns with previous reports describing non‐linear relationships between gene transcription and protein levels in hormonally responsive tissues (Liu et al. 2016; Vogel and Marcotte 2012).

These findings emphasise the importance of molecular analyses in understanding the functional dynamics of the kisspeptin system in the reproductive physiology of bitches. These data indicate that kisspeptin signalling plays an important role in local regulation within reproductive tissues. Moreover, the results of this study support the hypothesis that the kisspeptin system regulates not only within the central nervous system but also in reproductive tissues, contributing to physiological tissue adaptations during the oestrous cycle. Specifically, uterine *KISS1* expression peaked during estrus, whereas *Ki‐67* expression reached its highest levels during proestrus and remained elevated in estrus, supporting a role in endometrial proliferation and remodelling during the follicular phase. These results are consistent with the findings of Bozkurt et al. (2026), which demonstrated that KP‐10 administration in anestrus bitches stimulated gonadotropin secretion and influenced ovarian activity. Collectively, these results highlight the importance of kisspeptin signalling in coordinating systemic endocrine responses with local tissue changes across the oestrous cycle. Future studies in bitches aimed at elucidating the role of the kisspeptin system in reproductive organs may facilitate the development of therapeutic strategies for infertility.

## Author Contributions

Ö.K.A.: designed the study, supervision, project administration, performed molecular analyses, wrote the original draft, conducted the formal analysis, reviewed and edited the paper. A.R.A.: performed laparotomies and tissue sampling, performed molecular analyses and wrote the original draft. Ö.Ö.: performed histopathological and immunohistochemical analyses, reviewed the original draft. G.B.: performed laparotomies and tissue sampling, reviewed the original draft. Ş.T.: performed histopathological and immunohistochemical analyses.

## Funding

This work was supported by Burdur Mehmet Akif Ersoy University Scientific Research Project Unit (Project number: 0701‐MP‐21).

## Conflicts of Interest

The authors declare no conflicts of interest.

## Data Availability

The data that support the findings of this study are available from the corresponding author upon reasonable request.
